# Peripheral Hypoarousal but Not Preparation-Vigilance Impairment Endures in ADHD Remission

**DOI:** 10.1177/1087054717698813

**Published:** 2017-03-31

**Authors:** Sarah-Naomi James, Celeste H. M. Cheung, Anna-Sophie Rommel, Gráinne McLoughlin, Daniel Brandeis, Tobias Banaschewski, Philip Asherson, Jonna Kuntsi

**Affiliations:** 1 King’s College London, UK; 2 University of London, UK; 3 Heidelberg University, Germany; 4 University of Zurich, Switzerland

**Keywords:** ADHD, remission, EEG, arousal, adolescents

## Abstract

**Objective:** This study investigates whether impairments associated with persistent ADHD—impaired attention allocation (P3 amplitude), peripheral hypoarousal (skin conductance level [SCL]), and adjustment in preparatory state (contingent negative variation [CNV])—reflect enduring deficits unrelated to ADHD outcome or are markers of ADHD remission. **Method:** Young people with childhood ADHD (73 persisters and 18 remitters) and 144 controls were compared on neurophysiological measures during two conditions (baseline and fast-incentive) of a four-choice reaction time task. **Results:** ADHD remitters differed from persisters, and were indistinguishable from controls, on baseline P3 amplitude and fast-incentive CNV amplitude (*p* ≤ .05). ADHD remitters differed from controls (*p* ≤ .01), and were indistinguishable from persisters (*p* > .05), on baseline SCL. **Conclusion:** Preparation-vigilance measures were markers of ADHD remission, confirming previous findings with other measures. Yet, SCL-measured peripheral hypoarousal emerges as an enduring deficit unrelated to ADHD improvement. Future studies should explore potential compensatory mechanisms that enable efficient preparation-vigilance processes in ADHD remitters.

## Introduction

In many individuals with childhood ADHD, the symptoms and impairments persist into adolescence and adulthood (Faraone, Biederman, & Mick, 2006; Polanczyk, de Lima, Horta, Biederman, & Rohde, 2007; Simon et al., 2009). Yet others show significant improvement, such that they no longer obtain the diagnosis and appear free of clinically significant impairment (Faraone et al., 2006). By studying those whose ADHD improves over time, we can gain insight into the pathways to remission.

In our recent follow-up study from childhood to adolescence and early adulthood, ADHD persistence rate was 79% (Cheung et al., 2016; Cheung et al., 2015). We used cognitive and electroencephalography (EEG) and event-related potential (ERP) measures to investigate whether the cognitive-neurophysiological impairments associated with ADHD improve together with symptom improvement, or reflect enduring deficits. Data from a cued continuous performance task and an arrow flanker task identified measures of preparation-vigilance and error detection as markers of ADHD remission (Cheung et al., 2016; Michelini et al., 2016). These measures—reaction time variability (RTV), omission errors, congruent errors, ERPs of response preparation and error detection, delta and theta activity—showed impairments in ADHD persisters only, with ADHD remitters indistinguishable from controls. In contrast, measures of inhibition, working memory, speed of processing, and conflict monitoring were not sensitive to ADHD remission/persistence. Our results are in line with other recent studies that found executive control measures not being associated with ADHD remission (Biederman et al., 2009; McAuley, Crosbie, Charach, & Schachar, 2014; Pazvantoğlu et al., 2012; van Lieshout, Luman, Buitelaar, Rommelse, & Oosterlaan, 2013); yet this pattern was not observed in three other studies (Bédard, Trampush, Newcorn, & Halperin, 2010; Francx et al., 2015; Halperin, Trampush, Miller, Marks, & Newcorn, 2008).

Further candidates as markers of remission are other measures that show malleability in individuals with ADHD. Using the Fast Task, a four-choice reaction time task under two conditions (a slow, unrewarded baseline condition and a fast condition with rewards), we have studied the extent to which individuals with persistent ADHD can improve their performance and associated neurophysiological functions between the two conditions. The baseline condition of the Fast Task induced impairments in RTV, attention allocation (P3 amplitude), and hypoarousal (skin conductance [SC] level [SCL]) in adolescents and young adults with persistent ADHD, but each of these improved significantly between conditions in the persistent ADHD group, indicating malleability of these measures in individuals with ADHD (Cheung et al., 2017; James, Cheung, Rijsdijk, Asherson, & Kuntsi, 2016). In the fast-incentive condition, individuals with persistent ADHD were indeed now indistinguishable from controls on attention allocation (P3) and peripheral arousal (SCL), yet another impairment was still observed, as the participants with persistent ADHD, unlike controls, were not able to adjust their preparatory state (contingent negative variation [CNV] amplitude) in a changed context (Cheung et al., 2017; James et al., 2016).

Although our recent analyses indicated that RTV consistently emerges as a marker of remission across various tasks, the most robust effect was in the Fast Task (Cheung et al., 2016; Michelini et al., 2016). However, it is unclear whether other impairments that emerged on the Fast Task in ADHD persisters are similarly markers of ADHD remission, or reflects enduring deficits. Here, we compare the group differences between ADHD persisters, remitters, and controls on attenuated attention allocation (P3 amplitude) and peripheral hypoarousal (SCL) in the baseline condition, and attenuated preparatory state (CNV amplitude) in the fast-incentive condition of the Fast Task to investigate how these impairments relate to ADHD outcome.

## Method

### Sample

The sample consists of 279 participants, who were followed up on average 5.8 years (standard deviation [*SD*] = 1.1 years) after initial assessments: A total of 110 had a diagnosis of combined-type ADHD in childhood (10 sibling pairs and 90 singletons), and 169 were control participants (76 sibling pairs and 17 singletons). Full details on this sample can be found elsewhere (Cheung et al., 2016; Cheung et al., 2015). Briefly, participants with ADHD were initially recruited from specialized ADHD clinics (Kuntsi et al., 2010), and control participants from schools in the United Kingdom. Exclusion criteria at both assessments included IQ <70, autism, epilepsy, brain disorders, and any genetic or medical disorder associated with externalizing behaviors that might mimic ADHD. Among those with childhood ADHD, 87 (79%) continued to meet clinical (*DSM-IV*) levels of ADHD symptoms and impairment (ADHD “persisters”), whereas 23 (21%) were below the clinical cutoff (ADHD “remitters”; Cheung et al., 2016; Cheung et al., 2015). Almost half (47%) of the participants with childhood ADHD were being treated with stimulant medication at follow-up. Parents of all participants gave informed consent following procedures approved by the London-Surrey Borders Research Ethics Committee (09/H0806/58).

From the original follow-up sample, 252 participants (82 ADHD persisters, 18 ADHD remitters, 78 controls, and 74 control siblings) had SC measured (as SC data collection only started after initial participants had already been assessed). Due to SC equipment failure, 10 ADHD-persistent participants and eight control participants were excluded. For analyses, both members of control sibling pairs formed the control group (*n* = 144); siblings of ADHD probands were excluded unless they had an ADHD diagnosis themselves. The final sample consisted of 73 ADHD persisters (71 singletons and one sibling pair; mean age = 18.1 years, *SD* = 2.9 years), 18 ADHD remitters (18 singletons; mean age = 19.05 years, *SD* = 2.68 years), and 144 controls (72 sibling pairs; mean age = 17.3 years, *SD* = 2.15 years; Supplementary Material I). At follow-up, ADHD persisters, remitters, and controls differed in age and IQ, and there were significantly more males in the remitted group than in the other two groups (Supplementary Material I).

### Procedure

Participants were recontacted by telephone and scheduled for a follow-up clinical interview and a cognitive-EEG assessment with simultaneous SC assessment. The Fast Task was administered as part of a longer assessment session. For those prescribed stimulants, a 48-hr ADHD medication–free period was required. All participants were asked to abstain from caffeine, smoking, and drug and alcohol use on the day of testing, and subsequent adherence questions were asked on the day. Face-to-face or telephone clinical interviews were administered to the parent of each ADHD proband shortly before or after the participant’s assessment.

### Measures

#### IQ

The vocabulary and block design subtests of the Wechsler Abbreviated Scale of Intelligence (WASI; Wechsler, 1991) were administered to all participants to derive an estimate of IQ.

#### ADHD diagnosis

The diagnostic interview for ADHD in adults (DIVA; Kooij & Francken, 2007) was conducted by trained researchers with parents of the ADHD probands, to assess Diagnostic and Statistical Manual of Mental Disorders (4th ed.; *DSM-IV*; American Psychiatric Association, 1994) defined ADHD presence and persistence for the sample. Evidence of impairment commonly associated with ADHD was assessed with the Barkley’s Functional Impairment Scale (BFIS; Barkley & Murphy, 2006) during interviews with parents. Each item ranges from 0 (*never* or *rarely*) to 3 (*very often*). Participants were classified as “affected” at follow-up if they scored a “yes” on ≥six items in either the inattention or hyperactivity/impulsivity domains on the DIVA and if they scored ≥2 on two or more areas of impairments on the BFIS.

#### The Fast Task

The slow-unrewarded (baseline) condition followed a standard warned four-choice reaction time (RT) task (Andreou et al., 2007). A warning signal (four empty circles, arranged side by side) first appeared on the screen. At the end of the fore period (presentation interval for the warning signal), the circle designated as the target signal for that trial was filled (colored) in. Participants were asked to press the response key that directly corresponded to the position of the target stimulus. Following a response, the stimuli disappeared from the screen and a fixed intertrial interval of 2.5 s followed. Speed and accuracy were emphasized equally. If the child did not respond within 10 s, the trial terminated. To investigate the extent to which a response style characterized by slow and variable speed of responding can be maximally reduced, the task includes a comparison condition that uses a fast event rate (fore period of 1 s) and incentives. This condition started immediately after the baseline condition and consisted of 80 trials and a fixed intertrial interval of 2.5 s (Andreou et al., 2007; James et al., 2016; more detail can be found in Supplementary Material II). The fast-incentive condition is always administered after the baseline condition.

#### EEG recording and preprocessing

The EEG was recorded from 62 channel DC (direct current)-coupled recording system (extended 10-20 montage), with a 500 Hz sampling rate, impedances kept below 10 kO, and FCz as the reference electrode. The electrooculograms (EOGs) were recorded from electrodes above and below the left eye and at the outer canthi. The EEG data were analyzed using Brain Vision Analyser Version 2.0 (Brain Products, Germany). After down-sampling the data to 256 Hz, the EEG data were rereferenced to the average and filtered offline with digitally band-pass (0.1-30 Hz, 24 dB/oct) Butterworth filters. Ocular artifacts were identified from the data using independent component analysis (ICA). The extracted independent components were manually inspected, and ocular artifacts were removed by back-projection of all but those components. Data with other artifacts exceeding +100mV in any channel were rejected. All averages contained at least 20 sweeps. P3 amplitude was analyzed as the area amplitude measure (µV*ms) at Pz between 250 and 450 ms, to reduce bias due to the varying noise levels induced by the different task conditions (Luck, 2012). For the P3 analyses, all the accepted trials were baseline-corrected using a prestimulus baseline of 200 ms. The mean amplitudes of this pretarget period (−200 ms to 0 ms, using a technical zero baseline as in previous CNV work; Albrecht et al., 2013; Banaschewski et al., 2003) at Cz were also analyzed separately as a CNV measure. This short interval not only corresponded to the P3 baseline but also captured the short CNV in the fast-incentive condition with its 1-s cue target interval (Cheung et al., 2017).

#### Skin conductance (SC)

SC data were measured by attaching a pair of reusable 8 mm diameter silver-silver chloride electrodes on the palm of the hand (thenar eminence and hypothenar eminence) of participant’s nondominant hand at the start of the testing session. A nonsaline gel was used to increase impedance and help establish an electrical signal. A constant imperceptible voltage (0.5 V) was applied. SC was recorded using PSYCHLAB SC5 24 bit equipment system, which has an absolute accuracy of ±0.1 µS (PSYCHLAB, UK; James et al., 2016). SC data values were calculated using a SC system which is based on a SC sigmoid-exponential model that allows the tonic measure of SCL to be disentangled from phasic, stimulus-associated, skin conductance responses (SCRs), and further allows the decomposition of overlapping SCRs (Boucsein, 2012; Figner & Murphy, 2011; Lim et al., 1997; Williams et al., 2001). This system, therefore, is appropriate to use in conditions with long and short interstimulus intervals (Williams et al., 2000). The statistical model was applied to each condition, as a whole. Means of SCL were calculated per participant, across each condition (James et al., 2016).

### Statistical Analyses

Age was used as a covariate in all analyses. Analyses were initially performed without controlling for IQ, but we subsequently reran all analyses with IQ as a covariate to examine IQ effects. Gender was not included as a covariate in the group analyses to avoid controlling for ADHD status (Cheung et al., 2016; James et al., 2016; Michelini et al., 2016). Instead, we explored the effect of gender by rerunning all analyses with the females (*n* = 15) removed; the pattern of results remained the same (results are available from first author upon request). RTV and SCL data were skewed and transformed using the optimized minimal skew (lnskew0) command in Stata Version 11.1 (Stata Corporation, College Station, Texas). As these were sibling data, the data were analyzed using random intercept models and regression in Stata. The random intercept model is a multilevel regression model that can be used as an alternative to ANCOVA to control for genetic relatedness (where both siblings from a pair are included in analyses) in a repeated-measures design, using a “robust cluster” command to estimate standard errors (Cheung et al., 2016; Tye et al., 2012; Wood, Asherson, Rijsdijk, & Kuntsi, 2009). We first computed the main effects of group (ADHD persistent vs. ADHD remittent vs. controls), condition (baseline vs. fast-incentive), and group-by-condition interactions for all measures. Post hoc analyses were then conducted to investigate the differences between ADHD remitters and persisters, and controls. Means and *SD*s of measures in the baseline and fast-incentive condition are reported in Supplementary Material I. Cohen’s *d* effect sizes were calculated (Supplementary Material I), where 0.2 is considered a small effect, 0.5 a medium effect, and 0.8 a large effect. By controlling for differences in the baseline condition, we were additionally able to investigate whether groups differed in the slope from the baseline to fast-incentive condition, indexing the degree of change. Pearson correlations were also conducted on these measures to examine their associations with DIVA ADHD symptom scores, and clinical impairment within those who had a childhood ADHD diagnosis, with age and gender included as covariates.

## Results

The results for comparisons involving the ADHD-remittent group are new (apart from baseline RTV; Cheung et al., 2016) and are the focus of the present study. For ease of comparison and completeness, here we also report on the statistics from the ADHD-persistent and control comparisons, which have previously been reported (Cheung et al., 2017; James et al., 2016). However, the sample included in the current study is not exactly the same as reported in our previous studies, as we included only participants with complete SC measures.

### RTV

**Figure 1.**
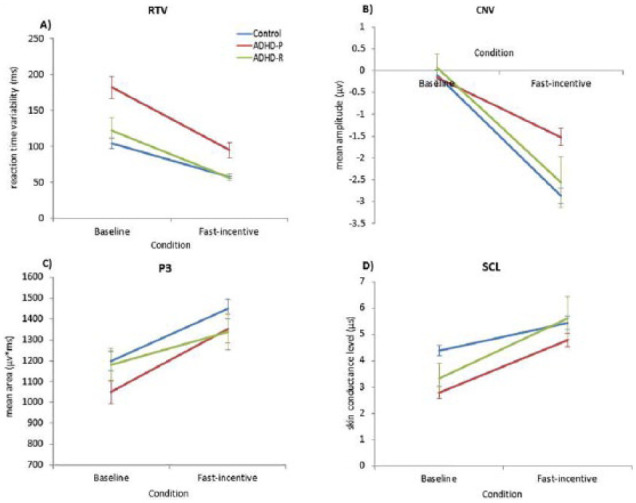
Group comparisons on (a) RTV, (b) CNV at Cz, (c) P3 amplitude at Pz, and (d) SCL across baseline and fast-incentive conditions of the Fast Task in ADHD remitters (ADHD-R; in green), ADHD persisters (ADHD-P; in red), and control participants (Controls; in blue). *Note.* Data from ADHD persisters and control participants in the full sample have already been presented for RTV, CNV, and P3 (Cheung et al., 2017), and SCL (James, Cheung, Rijsdijk, Asherson, & Kuntsi, 2016), but for ease of comparison, results specific to this analysis have been replicated here with the additional ADHD remitter group. RTV = reaction time variability; CNV = contingent negative variation; SCL = skin conductance level.

For RTV data, a random intercept model indicated a significant main effect of condition (*z* = −10.26, *p* < .01), main effect of group (*z* = 4.37, *p* < .01), but no main group-by-condition interaction (*z* = −0.73, *p* = .46; Figure 1a). Post hoc analyses revealed that, in the baseline condition, ADHD remitters had significantly decreased RTV compared with ADHD persisters (*t* = −2.49, *p* < .05, *d* = 0.79), but did not differ from controls (*t* = 1.21, *p* = .12, *d* = 0.17); ADHD persisters had significantly increased RTV compared with controls (*t* = 7.06, *p* < .05, *d* = 1.20). In the fast-incentive condition, ADHD remitters had significantly decreased RTV compared with ADHD persisters (*t* = −1.62, *p* < .05, *d* = 0.47) but did not differ from controls (*t* = 1.40, *p* = .10, *d* = 0.31; Figure 1a); ADHD persisters had significantly increased RTV compared with controls (*t* = 6.16, *p* < .05, *d* = 0.90). The within-group decrease from the baseline to fast-incentive condition was significant in ADHD remitters (*t* = −2.34, *p* < .05), ADHD persisters (*t* = −8.09, *p* < .05), and controls (*t* = −8.09, *p* < .05). The slope in RTV (indexing the degree of change from the baseline to the fast-incentive condition) in ADHD remitters was significantly less steep compared with ADHD persisters (*t* = −1.87, *p* = .05, *d* = 0.47), but was not significantly different compared with controls (*t* = 0.58, *p* = .56, *d* = 0.12). The slope in RTV was significantly greater in ADHD persisters compared with controls (*t* = −2.26, *p* < .05, *d* = 0.31).

### CNV Amplitude

**Figure 2.**
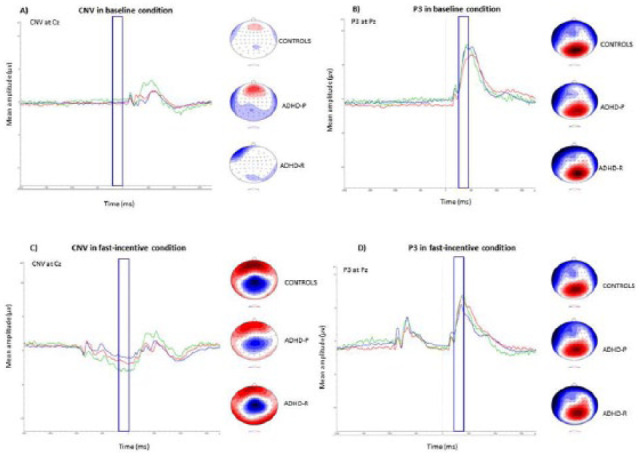
Grand averages for stimulus-locked ERPs of the CNV at Cz electrode between −200 and 0 ms (shown on the left), and of the P3 at Pz electrode between 250 and 450 ms (shown on the right), in both the (a and b) baseline and (c and d) fast-incentive conditions of the Fast Task in ADHD remitters (ADHD-R; in green), ADHD persisters (ADHD-P; in red), and control participants (Controls; in blue), with topographic maps. *Note.* Data from ADHD persisters and control participants in the full sample have already been presented for CNV and P3 (Cheung et al., 2017), but for ease of comparison, results specific to this analysis have been replicated here with the additional ADHD remitter group. ERP = event-related potential; CNV = contingent negative variation.

For CNV amplitude, a random intercept model indicated a significant main effect of condition (*z* = −15.37, *p* < .01), main effect of group (*z* = 2.59, *p* < .05), and a trend level significance of group-by-condition interaction (*z* = −1.66, *p* = .09; Figure 1b). Post hoc analyses revealed that, in the baseline condition, ADHD remitters did not differ in CNV amplitude compared with ADHD persisters (*t* = 0.57, *p* < .51) or controls (*t* = 1.17, *p* < .24; Figures 1b and 2a); ADHD persisters also did not differ in CNV amplitude compared with controls (*t* = 0.80, *p* = .21, *d* = 0.13). In the fast-incentive condition, ADHD remitters showed significantly increased CNV amplitude, compared with ADHD persisters (*t* = 2.44, *p* < .01, *d* = 0.74), but were not significantly different compared with controls (*t* = −0.12, *p* = .91, *d* = 0.02; Figures 1b and 2c); ADHD persisters had significantly decreased CNV amplitude compared with controls (*t* = 4.72, *p* < .05, *d* = 0.76). There was a significant within-group increase in CNV amplitude from the baseline to fast-incentive condition in ADHD remitters (*t* = 5.01, *p* < .01), ADHD persisters (*t* = 5.35, *p* < .05), and controls (*t* = 12.81, *p* < .05). In ADHD remitters, the slope in CNV amplitude (indexing the degree of change from the baseline to the fast-incentive condition) was significantly steeper compared with ADHD persisters (*t* = 3.25, *p* < .01, *d* = 0.88), but did not differ compared with controls (*t* = −0.79, *p* = .43, *d* = 0.19); the slope in CNV amplitude was significantly steeper in controls compared with ADHD persisters (*t* = 4.34, *p* < .01, *d* = 0.68).

### P3 Amplitude

For P3 amplitude, a random intercept model indicated a significant main effect of condition (*z* = 47.76, *p* < .01), but no main effect of group (*z* = −0.09, *p* = .92), or group-by-condition interaction (*z* = −0.24, *p* = .81; Figure 1c). Post hoc analyses revealed that, in the baseline condition, ADHD remitters showed significantly increased P3 amplitude compared with ADHD persisters (*t* = 3.51, *p* < .05, *d* = 0.56), but were not different compared with controls (*t* = −1.64, *p* = .12, *d* = 0.18; Figures 1c and 2b); ADHD persisters had significantly decreased P3 compared with controls (*t* = 1.88, *p* < .05, *d* = 0.30). In the fast-incentive condition, ADHD remitters were not significantly different in P3 amplitude compared with ADHD persisters (*t* = 1.22, *p* < .01, *d* = 0.20) or controls (*t* = −0.22, *p* = .81, *d* = 0.13; Figures 1c and 2d); ADHD persisters did not differ in P3 amplitude compared with controls (*t* = 1.20, *p* < .12, *d* = 0.31). There was a significant within-group increase in P3 amplitude from the baseline to fast-incentive condition in ADHD remitters (*t* = 23.44, *p* < .01), ADHD persisters (*t* = 26.84, *p* < .05), and controls (*t* = 32.90, *p* < .05). The slope in P3 amplitude between the baseline and fast-incentive condition in ADHD remitters was significantly less than in ADHD persisters (*t* = 2.22, *p* < .05, *d* = 0.57), but did not differ from controls (*t* = 1.51, *p* = .13, *d* = 0.31); the slope in P3 amplitude was significantly greater in ADHD persisters, compared with controls (*t* = 1.45, *p* < .05, *d* = 0.31).

### SCL

For SCL data, a random intercept model indicated a significant main effect of condition (*z* = 25.43, *p* < .01), a significant group-by-condition interaction (*z* = 2.33, *p* < .05) but no significant main effect of group (*z* = −0.34, *p* = .73; Figure 1d). Post hoc analyses revealed that, in the baseline condition, ADHD remitters did not differ from ADHD persisters (*t* = −0.52, *p* = .61, *d* = 0.15), but had decreased SCL compared with controls (*t* = −3.70, *p* < .01, *d* = 0.89; Figure 1d). In the fast-incentive condition, no group differences emerged between ADHD remitters and ADHD persisters (*t* = 0.23, *p* = .81, *d* = 0.08), or between ADHD remitters and controls (*t* = 0.30, *p* = .77, *d* = 0.09). Analyses between ADHD persisters and controls in the identical sample have previously been reported: ADHD persisters had significantly decreased SCL compared with controls in the baseline condition (*t* = −5.64, *p* < .001), but the groups did not differ in the fast-incentive condition (*t* = 1.10, *p* = .27; James et al., 2016). The within-group increase in SCL from the baseline to fast-incentive condition was significant in ADHD remitters (*t* = 8.86, *p* < .01). Significant within-group increases in SCL, from the baseline to fast-incentive condition, in ADHD persisters and controls have previously been reported (*t* = 7.52, *p* < .01, *t* = 6.44, *p* < .01, respectively; James et al., 2016). The slope in SCL between the baseline and fast-incentive condition in ADHD remitters did not differ from ADHD persisters (*t* = 0.20, *p* = .84, *d* = 0.06) or controls (*t* = 1.03, *p* = .31, *d* = 0.24); the slope in SCL was steeper in ADHD persisters, compared with controls (*t* = 1.94, *p* < .05, *d* = 0.31).

The analyses were rerun separately with the following adjustments using IQ as a covariate, using a male-only sample, and using indicated drug and alcohol use as covariates; the significance of results remained unchanged (results available from first author by request).

### Associations With the Continuums of ADHD Symptoms and Impairments

In those with childhood ADHD (*n* = 91), ADHD impairment at follow-up correlated significantly with baseline RTV and P3 amplitude, and with CNV amplitude in the fast-incentive condition (Table 1). The only significant correlation with ADHD symptoms was observed for RTV in the baseline condition, as reported previously for the full follow-up sample of those with childhood ADHD (*n* = 110; Cheung et al., 2016). No other significant associations were observed.

**Table 1.** Pearson Correlations (Two-Tailed) of Cognitive Performance (RTV), ERP (CNV Amplitude and P3 Amplitude), and Skin Conductance (SCL) Measures With Interview-Based DIVA ADHD Symptoms and BFIS Clinical Impairment Within the ADHD Group Only (*n* = 91), Without Controlling for IQ.

	ADHD symptoms	Impairment
	*r*	*r*
Baseline condition		
RTV	.20^a^*	.27^a^*
CNV	.20	.05
P3	−.16	−.36*
SCL	.01	−.18
Fast-incentive condition		
RTV	.13	.15
CNV	.18	.30*
P3	−.11	−.02
SCL	−.06	−.10

*Note.* Data from RTV in the baseline condition in the full sample have already been reported (Cheung et al., 2016), but for ease of comparison, results have been replicated here in the subsample. CNV amplitude at Cz, P3 amplitude at Pz. RTV = reaction time variability; ERP = event-related potential; CNV = contingent negative variation; SCL = skin conductance level; DIVA = diagnostic interview for ADHD in adults; BFIS = Barkley’s Functional Impairment Scale.

a Denotes this correlation has previously been reported in the full sample (Cheung et al., 2016).

* *p* < .05.

## Discussion

We have previously linked persistent ADHD to impaired attention allocation (P3 amplitude) and peripheral hypoarousal (SCL) during baseline reaction time performance, as well as to an inability to adjust the preparatory state in a changed context (CNV amplitude in a fast condition with incentives; Cheung et al., 2017; James et al., 2016). In a comparison between ADHD persisters, ADHD remitters, and controls on these neurophysiological indices, we now find that P3 amplitude and CNV amplitude are markers of remission, consistent with previously reported findings for RTV and other markers of preparation-vigilance (Cheung et al., 2016; Michelini et al., 2016). In contrast, hypoarousal, as measured with SC during baseline RT performance, emerges as an enduring deficit, that is unrelated to ADHD symptom improvement.

The finding of SCL-indexed hypoarousal reflecting an enduring impairment in the baseline condition is therefore not mirroring the remission pattern observed for RTV as expected, because we have previously found a link between SCL-indexed hypoarousal and RTV in individuals with persistent ADHD, under identical testing conditions (James et al., 2016). Overall, these data suggest that, as ADHD remitters show peripheral underarousal during baseline RT performance, improved arousal regulation does not account for the strong, control group–level cognitive-EEG performance now observed among the ADHD remitters. The current findings therefore indicate that decreased SCL-measured peripheral hypoarousal is not associated with ADHD symptom improvement in ADHD remitters, and is unlikely to be a suitable treatment target. Future research should investigate potential compensatory processes to better understand the pathways of improved attentional performance in ADHD remitters, despite SCL-measured peripheral hypoarousal.

Analyses on continuous measures of ADHD outcome further confirmed the lack of an association between SC measures of arousal and either ADHD symptoms or impairment at follow-up. The ERP markers of remission in the group analyses—P3 in the baseline condition and CNV in the fast-incentive condition—were significantly associated with the continuous impairment scores, though only RTV was significantly associated with both ADHD symptoms and impairment (Cheung et al., 2016).

Although the Fast Task paradigm is not ideal for measuring CNVs due to the different interstimulus intervals, in the fast-incentive condition, where group differences between ADHD remitters and ADHD persisters emerged, we observed a typical CNV distribution in all groups (Figure 2c), suggesting that the CNV is a sensitive marker in this condition. The main limitation of our study, due to the quasi-experimental design, is the modest number of remitters, which means we cannot run more complex multivariate analyses across variables, and indicates that our findings require future replication before any stronger inferences are drawn. Furthermore, we had a male-only remittent group, making it unfeasible to investigate whether there are differences in cognitive-neurophysiological measures between male and female individuals with remittent ADHD. As our sample involved adolescents and young adults who are still undergoing cortical development, future follow-up studies when all participants have reached adulthood will be beneficial to further elucidate developmental trajectories.

Overall, our results indicate an enduring deficit in peripheral hypoarousal during baseline RT performance in ADHD remitters, whereas preparation-vigilance processes (P3 amplitude in the baseline condition and CNV amplitude in the fast-incentive condition, (as well as RTV Cheung et al., 2016) are markers of remission. This indicates there may be alternative compensatory mechanisms to counteract the peripheral hypoarousal in ADHD remitters. Yet peripheral hypoarousal is context-dependent, rather than a stable deficit, in ADHD remitters as they, similar to ADHD persisters (James et al., 2016), were indistinguishable from controls on SCL in the faster condition with rewards. Future studies should aim to explore potential compensatory mechanisms that enable efficient preparation-vigilance processes, even in task conditions that induce persisting hypoarousal, in ADHD remitters.
